# Knowledge, views, practice and self-efficacy regarding copper intrauterine contraceptive device usage among public primary care doctors in Sarawak: A cross-sectional study

**DOI:** 10.51866/oa.662

**Published:** 2025-04-28

**Authors:** Yik Huang Chua, Chare Chee Kueh, Sing Seng Lee, Li Lian Lim, Kii Han Sia, Hie Ping Sii, Ishak Maziah, Chandramani Thuraisingham, Cheong Lieng Teng

**Affiliations:** 1 MBBS, MFamMed, FRACGP, Professor, Department of Family Medicine, IMU University, Seremban Campus, Malaysia. Email: tengcl@gmail.com; 2 MD, icFRACGP, General Practitioner, Taupo Health Centre, New Zealand.; 3 MBBS, Medical Officer, Klinik Kesihatan Sarikei, Sarawak, Malaysia.; 4 MD, MAFP/icFRACGP, Family Medicine Specialist, Klinik Kesihatan Tatau, Sarawak, Malaysia.; 5 MD, MAFP/icFRACGP, General Practitioner, Australia.; 6 MBBS, MAFP/icFRACGP, Family medicine Specialist, Klinik Kesihatan Selangau, Sarawak.; 7 MMed (Fam Med) UKM, Family Medicine Specialist, Klinik Kesihatan Asajaya, Sarawak.; 8 MBBS, FRACGP, FAFP, MHPE, AM, DRM, PG DOH, Clinical Professor, Department of Family Medicine, IMU University, Seremban Campus, Malaysia.

**Keywords:** Contraception, Health Knowledge, Attitudes and Practice, Intrauterine Devices, Malaysia, Physicians, Primary Care, Sarawak

## Abstract

**Introduction:**

Intrauterine contraceptive device (IUCD) insertion is a reversible and effective way to reduce unplanned pregnancy. Doctors play a key role in IUCD usage. This study aimed to assess knowledge, views, practice and self-efficacy regarding copper IUCD usage among public primary care doctors in Sarawak.

**Methods:**

A cross-sectional study was conducted in all public primary care clinics in Sarawak. A selfadministered online survey form was distributed to doctors in the eligible clinics.

**Results:**

A total of 312 doctors participated in the survey. Most of them (81.4%) worked in a clinic where IUCDs were available. The median knowledge score among the medical officers was 15 (maximum score: 20). The knowledge score was correlated with the length of primary care experience and was higher among the doctors who had prior IUCD training and ever had inserted IUCDs. The doctors were concerned about the side effects of IUCDs, especially perforation and pelvic inflammatory diseases, and were negatively influenced by the additional counselling time required when recommending IUCDs. They recognised IUCD usage as a long-term and reversible contraception method. Most (69.2%) doctors had inserted IUCDs before, but only 45.9% had inserted five or more IUCDs. The self-efficacy of the doctors regarding IUCD insertion was moderate (63% of the total score).

**Conclusion:**

The knowledge and self-efficacy of the public primary care doctors in Sarawak were moderate. Although IUCDs were widely available, the number of IUCD insertions was relatively small. Further research is warranted to quantify IUCD usage and identify its barriers among nurses and patients.

## Introduction

Globally, among 1.9 billion women of reproductive age (15-49 years), approximately 874 million use a modern contraceptive method.^1^ The National Health and Morbidity Survey (NHMS) 2022 revealed a 42.8% prevalence rate of contraception among women of reproductive age in Malaysia and 34.5% and 45.2% prevalence rates of modern contraception in Malaysia and Sarawak, respectively.^2^ The NHMS 2022 also showed oral contraceptive pills (33.1%), injectable contraceptives (16.4%) and coitus interruptus (9.6%) as the top three contraceptive methods used in Malaysia and indicated a relatively low (6.2%) prevalence of intrauterine contraceptive device (IUCD) usage.^2^ A survey on contraceptive use among women of reproductive age in Samarahan, Sarawak, showed that the prevalence of contraceptive use was 43%, and among these users, IUCD usage was reported by only 3.8%.^3^

The use or non-use of IUCDs is influenced by many factors among providers and clients.^4,5^ IUCD implantation must be performed by skilled practitioners; thus, doctors play an important role in the utilisation of IUCDs among women. In their study exploring knowledge, attitudes and perceptions towards IUCD usage among public and private doctors in Kuala Lumpur, Chew et al. identified some gaps particularly in knowledge and perceptions.^6^ The current study aimed to determine whether similar issues exist among public primary care doctors in Sarawak.

## Methods

### Study setting and participants

A cross-sectional study was conducted in all public primary care clinics in Sarawak from August to December 2020. All doctors working in 122 public primary care clinics in Sarawak were invited to participate via WhatsApp messages facilitated by the State Health Department.

### Sample size and sampling

The total number of medical officers working in the target public healthcare clinics in Sarawak was estimated to be 450 in 2019. Universal sampling was utilised; hence, sample size estimation was not performed.

**Instrument**

The questionnaire for this study was adopted from the study by Chew et al.^6^ The questionnaire had six sections: (a) provider characteristics, (b) provider training in IUCD usage, (c) knowledge, (d) practice, (e) views and (f) self-efficacy, with a total of 46 questions. The Cronbach’s a value of the entire questionnaire was 0.769.^6^ The knowledge section included 20 questions: three multiple-choice questions and 17 statement-based questions, where participants were required to indicate ‘true/false’ or ‘yes/no’ as their answer. The knowledge score was computed by giving 1 point for each correct answer, with a maximum possible score of 20. The practice section assessed medical officers’ experience on IUCD insertion and the number of IUCDs inserted. Their views were explored through closed-ended questions on factors that promote or prevent their decision to recommend IUCDs. For the self-efficacy section, a 5-point Likert scale *(strongly agree, agree, unsure, disagree and strongly disagree)* was used to assess medical officers’ perceptions in providing IUCD services. The negatively worded statements (items 3-5) were reverse-scored to align with a unidirectional focus on positive self efficacy. The self-efficacy score was computed by summing the scores for the six items after reverse-coding the three negatively worded statements. The total possible self-efficacy score was 30. The questionnaire was administered as an online survey via Google Forms. The survey link was sent via WhatsApp, and three email reminders were sent at 1-month intervals.

### Data analysis

Data were analysed using IBM SPSS Statistics for Windows, version 28 (IBM Corp., Armonk, N.Y., USA). The main outcome measures were the knowledge, views, practice and self-efficacy of participants regarding IUCD usage. As the continuous variables such as age, the knowledge score and the self-efficacy score were not normally distributed, medians (interquartile ranges [IQRs] at 25^th^ and 75^th^ percentiles) were reported. Differences in the continuous variables (between two groups) were evaluated using a non-parametric test (Mann-Whitney U test). Linear correlations between two continuous variables were analysed using Spearman’s correlation. The statistical significance level was set at P<0.05.

## Results

A total of 450 doctors were invited to participate in the study, among whom 326 were finally included, yielding a response rate of 72%. Most of the participants were medical officers (n=312, 95.7%), while the rest were family medicine specialists (n=14; the data of this group were excluded from further analysis). The medical officers’ sociodemographic data and training in IUCD usage are shown in Table 1. The median age of the participants was 30 years. The median working duration in the designated clinic was 24 months. Most medical officers (81.4%) worked in a clinic where IUCDs were available. Two-thirds of the medical officers reported at least one IUCD insertion per month.

**Table 1:** Medical officers’ sociodemographic data and learning experience.

Variable	n (%) or median (IQR^*^)
Age, median (IQR) year	30 (29, 30)
Sex	
Male	105 (33.7)
Female	207 (66.3)
Primary care working experience, median (IQR) month	24 (12, 36)
IUCD available in the clinic	254 (81.4)
IUCD inserted per month	206 (66.0)
Training in IUCD insertion	194 (62.2)
Awareness of the WHO eligibility criteria for contraceptive use	142 (45.5)
Total	312

* IQR, interquartile range

### Knowledge regarding copper IUCD usage

The 20 questions assessing the medical officers’ knowledge with correct answers are shown in Table 2. In three knowledge items (items 3, 7 and 14), correct answers were provided by less than 50% of the participants. The median (IQR) knowledge score was 15 (13, 16). A total of 303 (97.1%) participants scored ≥10 points, while 180 (57.7%) participants scored ≥15 points. The knowledge score between the female and male medical officers did not significantly differ (Mann-Whitney U test, P=0.267). The knowledge score was correlated with the length of primary care experience (Spearman’s correlation, p=0.250, P<0.001). Additionally, the knowledge score was higher among the medical officers who had prior IUCD training and ever had inserted IUCDs (Mann-Whitney U test, P<0.001).

**Table 2:** Knowledge questions regarding copper IUCD usage and individual question scores of the participants.

Item	Answer key	Correct answer, n (%)
1. The copper IUCD can be used as an emergency contraception.	Yes	209 (67.0)
2. What is the maximum length of time a woman can use the multiload Cu 375 IUCD after it is inserted? (multiple options)	5 years	272 (87.2)
3. What is the effectiveness of the IUCD as measured by the pregnancy failure rate per 100 women-years of use? (multiple options)	0.2	131 (42.0)
4. How quickly does fertility typically return after removal of a copper IUCD? (multiple options)	Immediately	253 (81.1)
5. Do IUCDs increase a patient’s overall risk of ectopic pregnancy as compared to no contraception?	No	168 (53.8)
6. When can the IUCD be inserted provided it is reasonably certain the woman is not pregnant? Anytime during the menstrual cycle	True	208 (66.7)
7. When can the IUCD be inserted provided it is reasonably certain the woman is not pregnant? Within 48 hours postpartum	True	141 (45.2)
8. When can the IUCD be inserted provided it is reasonably certain the woman is not pregnant? Up to 7 days postpartum	False	218 (69.9)
9. When can the IUCD be inserted provided it is reasonably certain the woman is not pregnant? 4 weeks after delivery	True	286 (91.7)
10. What are the side effects or adverse outcomes associated with using a copper IUCD? Headache	No	255 (81.7)
11. What are the side effects or adverse outcomes associated with using a copper IUCD? Ectopic pregnancy	Yes	214 (68.6)
12. What are the side effects or adverse outcomes associated with using a copper IUCD? Increased weight gain	No	285 (91.3)
13. What are the side effects or adverse outcomes associated with using a copper IUCD? Migration	Yes	229 (73.4)
14. IUCDs can be inserted as a contraception in the following situation: History of PID a year ago, currently asymptomatic	Yes	152 (48.7)
15. IUCDs can be inserted as a contraception in the following situation: Recent spontaneous first-trimester abortion	Yes	216 (69.2)
16. IUCDs can be inserted as a contraception in the following situation: History of ectopic pregnancy	Yes	177 (56.7)
17. IUCDs can be inserted as a contraception in the following situation: Breastfeeding	Yes	297 (95.2)
18. IUCDs can be inserted as a contraception in the following situation: Diabetes mellitus	Yes	297 (95.2)
19. IUCDs can be inserted as a contraception in the following situation: Ischaemic heart disease	Yes	285 (91.3)
20. IUCDs can be inserted as a contraception in the following situation: Ischaemic heart disease	Yes	294 (94.2)

### Views and practice concerning IUCD usage

As demonstrated in Table 3, the medical officers were concerned about the potential side effects of IUCD usage (e.g. perforation and pelvic inflammatory diseases) and were negatively influenced by the additional counselling time required when recommending IUCDs. However, they recognised IUCD usage as a long-term and reversible contraceptive method.

At least two-thirds of the medical officers had inserted IUCDs before, and almost half of them had previously inserted at least five IUCDs, with the majority having done so within the past 6 months.

**Table 3:** Medical officers’ views and practice regarding copper IUCD insertion.

Variable	n (%)
**Views**	
Side effects that prevent me from recommending IUCDs	
Risk of perforation	201 (64.4)
Heavy menstrual bleeding	149 (47.8)
Increased risk of malignancy	29 (9.3)
Increased risk of ectopic pregnancy	127 (40.7)
Increased risk of pelvic inflammatory diseases	169 (54.2)
Factors that negatively influence my decisions to recommend IUCDs	
Unavailability or inadequate supply in the clinic	121 (38.8)
Need for additional time for counselling	164 (52.6)
Expensive cost	27 (8.7)
Too much side effects for patients	26 (8.3)
Preference for other methods	64 (20.5)
Factors or characteristics of IUCDs that encourage me to recommend to patients	
Long-term nature	297 (95.2)
Reversibility	237 (76.0)
Promotion by the Ministry of Health	93 (29.8)
Profitability	41 (13.1)
Effectiveness	207 (66.3)
**Practice**	
Ever inserted IUCDs	216 (69.2)
Number of IUCDs ever inserted	
<5	56 (25.9)
5-10	33 (15.3)
>10	66 (30.6)
Not sure	61 (28.2)
Last IUCD insertion^*^	
Within the last 6 months	118 (54.9)
6-12 months ago	52 (24.2)
More than a year ago	45 (20.9)

* One participant did not report this information.

### Self-efficacy in IUCD insertion

The medical officers’ self-efficacy in inserting IUCDs is presented in Table 4. Of the six items, the median score for the three of them fell within the *unsure* category, while that for the other three was in the *agree* category. The median self-efficacy score was 19 (IQR: 18, 21), representing 63% of the total score. There was no linear correlation between the knowledge score and selfefficacy score (p=0.076, P=0.179).

**Table 4:** Medical officers’ self-efficacy in IUCD insertion.

Item	Median (IQR)^*^
1. I feel confident that I can insert the IUCD with little difficulty to patients.	3 (3, 4)
2. I feel comfortable explaining IUCD issues to my patients.	4 (4, 4)
3. IUCDs are difficult to insert.	3 (3, 4)
4. It is difficult to convince patients that many rumours about the IUCD are actually false.	3 (2, 4)
5. The risk of complication when inserting an IUCD is too great.	4 (3, 4)
6. Providing IUCD insertion services is a good use of my skills and experience.	4 (4, 4)

* 1=strongly disagree, 2=disagree, 3=unsure, 4=agree, 5=strongly agree

## Discussion

In this study, the knowledge of the medical officers from the target public primary care clinics in Sarawak was relatively good (75%, median score: 15/20). Their views were mostly positive towards IUCD usage, and their self-efficacy in inserting IUCDs was moderate (63%, median score: 19/30). IUCDs were available in most public primary care clinics in Sarawak, and more than two-thirds of the medical officers had inserted at least one IUCD before. When we compare the proportion of the medical officers with a knowledge score of ≥15 in our study to that in the study by Chew et al.^6^ (57.7% vs 39.2%), the difference is statistically significant (χ^2^=18.62, P<0.001). This may be explained by the large proportion of private practitioners included in the study by Chew et al. (32.9%), who had lower knowledge scores.^6^

IUCD usage as a contraceptive method in Malaysia is relatively low (6.2%), in comparison with other contraceptive methods such as oral contraceptive pills.^2^ The corresponding figure in Sarawak is even lower (3.8%), as shown by Jawa and Rahman.^3^ The knowledge, views, practice and self-efficacy among the medical officers in our study may not adequately explain the low IUCD usage in Sarawak.

Surveys in other Asian countries showed that IUCD usage was much higher in certain countries (e.g. China [40.8%] and Vietnam [31.0%]).^7^ In their review, Najafi-Sharjabad et al. identified multiple barriers to the adoption of modern contraception, including cultural attitudes, lack of knowledge of methods and reproduction, sociodemographic factors and health service-related factors.^8^ Many providers have low or uneven levels of knowledge on IUCD and limited training. According to Daniele et al., misconceptions occur among both providers (e.g. fear of pelvic inflammatory diseases) and the lay public (e.g. fear of infertility or concerns about the insertion and removal processes and the effect on menses).^4^ The low uptake of IUCDs among Sarawakian women may also be due to their suboptimal knowledge, perception and acceptance of this contraceptive method.

Our study points to the need for further inservice education to address the indications, contraindications, side effects and misconceptions among medical officers, especially to improve the procedural skills of less experienced practitioners. In addition, the lack of research on the awareness and views of Sarawakian women on modern contraceptive methods, especially

IUCD implantation, suggests the need for both quantitative and qualitative research in the local setting.

### Study limitations

Our sample included only public primary care doctors; hence, the data cannot be generalised to public health nurses or healthcare providers working in private settings or hospitals. Additionally, the data were collected via an online survey; the lack of supervision and observation during administration could have introduced potential biases.^9^

## Conclusion

The knowledge and self-efficacy of the public primary care doctors in Sarawak were moderate. Most doctors worked in clinics providing IUCD services, but the number of IUCD insertions was relatively small. Further studies are warranted to quantify IUCD usage and determine its barriers among nurses and patients.

## Data sharing statement

Data are available upon request.

**How does this paper make a difference in general practice?**

- The knowledge and self-efficacy of public primary care doctors in Sarawak appear to be moderate.
- Most public primary care doctors are willing to provide IUCD services, but the number of IUCD insertions is relatively small due to various reasons.
- Further research is warranted to quantify IUCD usage and identify its barriers among nurses and patients in both public and private settings.
